# A comparative cost-effectiveness analysis of mechanical and pharmacological VTE prophylaxis after lower limb arthroplasty in Australia

**DOI:** 10.1186/s13018-019-1124-y

**Published:** 2019-04-02

**Authors:** Rafael Torrejon Torres, Rhodri Saunders, Kwok M. Ho

**Affiliations:** 1 Coreva Scientific, Kaiser-Joseph-Strasse 198-200, 79098 Freiburg, Germany; 2 Department of Intensive Care Medicine, Royal Perth Hospital, Perth, Western Australia Australia; 3 School of Population and Global Health, University of Western Australia, Perth, Western Australia Australia; 4 School of Veterinary and Life Sciences, Murdoch University, Perth, Western Australia Australia

**Keywords:** IPC, Australia, VTE, Arthroplasty, Oral anticoagulant

## Abstract

**Background:**

Venous thromboembolism (VTE) is a complication following surgery. Low-molecular-weight heparin (LMWH) or direct oral anticoagulants (DOACs) are efficacious but come with inherent bleeding risk. Mechanical prophylaxis, such as intermittent pneumatic compression (IPC), does not induce bleeding but may be difficult to implement beyond the immediate post-operative period. This study compared the cost and quality-adjusted life years (QALYs) saved of commonly used VTE prophylaxis regimens after lower limb arthroplasty.

**Methods:**

A previously published cost-utility model considering major efficacy and safety endpoints was updated to estimate the 1-year cost-effectiveness of different VTE prophylaxis regimens. The VTE strategies assessed included apixaban, dabigatran, rivaroxaban, LMWH, IPC, IPC + LMWH and IPC + apixaban. Efficacy data were derived from studies in PubMed, and cost data came from the 2017 Australian AR-DRG and PBS pricing schemes.

**Results:**

Costs for VTE prophylaxis including treatment of its associated complications over the first year after surgery ranged from AUD $644 (IPC) to AUD $956 (rivaroxaban). Across 500 simulations, IPC was the cheapest measure in 73% of simulations. In 97% of simulations, a DOAC was associated with the highest resulting QALYs. Compared to IPC, apixaban was cost-effective in 76.4% of simulations and apixaban + IPC in 87.8% of simulations. For VTE events avoided, the DOACs and IPC were on par. LMWH and LMWH + IPC were negatively dominated.

**Conclusions:**

Apixaban, IPC or a sequential/simultaneous combination of both is currently the most cost-effective VTE prophylaxis regimens. The choice between them is best guided by the relative VTE and bleeding risks of individual patients.

## Background

Venous thromboembolism (VTE) is a severe complication that can impact recovery after surgery. VTE mainly presents as either deep vein thrombosis (DVT) or pulmonary embolism (PE). From 2003 to 2010, VTE was the focus of an Australian governmental program to increase patient safety [1]. In 2009, VTE prophylaxis was included in the indicator catalogue for quality of care in Australia [2]. Despite this, the Organisation for Economic Co-operation and Development (OECD) reported that during the period of 2012–2013, DVT and PE rates following total hip and total knee arthroplasty (THA and TKA, respectively) procedures in Australia were above the OECD average, with the rate of DVT in Australia more than twice the OECD average [3].

There is a high burden of disease associated with VTE. In 2013, as part of the World Health Organization (WHO) patient safety program, Jha et al. showed that VTE is a major contributor to the loss of disability-adjusted life years attributable to unsafe or suboptimal medical care [4]. The financial burden of VTE reported by the Australian National Safety and Quality Health Service was AUD $1.72 billion per year [5]. Peel et al. estimated in 2015 that each year, approximately AUD $66 million of direct hospital costs are attributable to VTE after THA and TKA [6]. There is evidence that these costs can be reduced, and a 2013 assessment by Duff et al. found that increased adherence to evidence-based VTE prophylaxis regimens could substantially reduce the costs of care for surgical and high-risk medical patients [7]. This is of national importance given that each year over 100,000 THA and TKA procedures are performed in Australia [8, 9].

The evidence-based VTE prophylaxis called for by Duff et al. is usually found in the clinical guidelines. The utility of a national guideline on VTE prophylaxis in Australia has been called into question [10] and was rescinded in 2016 [11]. A number of local and association guidelines are though available [5, 12, 13]. In general, VTE prophylaxis after arthroplasty is recommended for 28–35 days after surgery and uses either anticoagulants and/or mechanical prophylaxis. A variety of anticoagulants are indicated, including low-molecular-weight heparin (LMWH) and the direct oral anticoagulants (DOACs): apixaban, dabigatran and rivaroxaban [5]. For mechanical prophylaxis, sequential or intermittent pneumatic compression (IPC) to the lower limbs is recommended. Studies have shown IPC to be equally effective as heparin for VTE prophylaxis, but with no inherent bleeding risk [14, 15]. Conversely, all anticoagulants by design have an inherent risk of bleeding. The risk of bleeding is hard to predict [16]. Bleeding events after major surgery can have serious and substantial clinical consequences, impacting on both patient quality of life and total healthcare costs, and hence these events must be balanced against the anticoagulants’ benefits for VTE prevention [10, 15].

Although multiple studies on the efficacy of different VTE prophylaxis regimens have been reported, there is currently no direct economic comparison between IPC, DOAC and LMWH. We hypothesised that due to different risk and benefit profiles of each VTE prophylaxis regimen, it is possible that some regimens are more favourable in its overall cost and benefits. In this analysis, we aimed to provide a comparison of the economics and outcomes of the VTE prophylaxis regimens commonly used in Australia after lower limb arthroplasty.

## Methods

A previously published cost-utility model specific to hip and knee arthroplasty was extended to consider short-term (1 year) health and economic outcomes associated with all the major available types of prophylaxis [17]. The published semi-Markov model was informed by a structured literature review to identify data related to THA and TKA, secondary outcomes and prophylaxis regimens. The following secondary safety outcomes were identified and accounted for pulmonary embolism (PE), DVT, post-thrombotic syndrome (PTS), heparin-induced thrombocytopenia (HIT), major and minor bleeding and intracranial haemorrhage (ICH) [17]. All patients started in post-surgical ‘No VTE’ state and received prophylaxis for 30 days. Progression between states was informed by LMWH-specific efficacies derived from the structured literature search.

The model was updated to use data specific to the Australian setting, and this included data on bleeding, VTE, HIT and PTS incidence. Costs used are from the 2017 PBS and AR-DRG pricing scheme. The payer perspective is taken and costs were adjusted for inflation to 2017 values using the ‘health’ consumer price index. No discounting was applied as the time horizon was only 1 year. Quality of life utilities were taken from studies reporting EuroQoL 5-dimensions (EQ-5D) scores, and utilities were considered to be additive. Base case parameters are presented in (Table 1).

**Table 1:** Base case parameters

Parameter	Value
Age	67.5 years [27]
Gender	40.5% [27]
Body mass index	31 kg/m^2^ [6]
History of VTE	7.7% [28]
Fraction THA	43.5% [8]
Fraction TKA	56.5% [9]
DVT incidence with LMWH	4.48% per 11.52 days [27]
PE incidence with LMWH	0.25% per 11.52 days [27]
Non-major bleed incidence with LMWH	9.9% per 12 days [28]
Major bleed incidence with LMWH	1.9% per 10 days [7]

*DVT* deep vein thrombosis, *LMWH* low-molecular-weight heparin, *PE* pulmonary embolism, *THA* total hip arthroplasty, *TKA* total knee arthroplasty, *VTE* venous thromboembolism

The VTE prophylaxis regimens considered as alternatives to LMWH are all approved DOACs in Australia: apixaban, dabigatran and rivaroxaban [5], as well as IPC alone (no added pharmacological anticoagulant, with supplemental low-dose aspirin) and in combination with LMWH or apixaban: IPC for 7 days followed by LMWH or apixaban. These VTE prophylaxis modalities were assessed using their risk ratios for events relative to LMWH, mostly derived from meta-analyses [14, 18–20]. The outcomes were the mean cost of care per patient and the mean quality-adjusted life years (QALYs) accumulated per patient for each modality. The incremental cost-effectiveness ratio (ICER) and cost per VTE avoided were then calculated. As the only mechanical method of VTE prophylaxis, each anticoagulant was compared to IPC alone.

To account for uncertainties in input parameters, sensitivity analysis with seeded uniform sampling over 500 runs was conducted. The results were presented as median savings with 95% credible interval (CrI). The number of simulations showing cost-effectiveness or dominance compared to the comparator was calculated with a willingness-to-pay threshold of AUD $50,000 per QALY gained and AUD $2750 per VTE avoided (5.5% [21] of the value applied to 1 year of perfect quality of life).

## Results

The cost of post-surgical care for VTE for 1 year ranged from AUD $644 to AUD $956 with costs lowest with IPC and highest with rivaroxaban. Results for QALYs were much more closely aligned between interventions, ranging from 0.8354 to 0.8429. Apixaban accumulated the most QALYs and IPC + LMWH the least. When compared to IPC alone, both LMWH and IPC + LMWH were dominated negatively, having a higher cost of care and accumulating fewer QALYs (Table 2). Use of DOACs increased both costs and QALYs compared to IPC, with their base-case ICER ranging from AUD $12,656 to AUD $55,714 per QALY gained (Table 2).

**Table 2:** Model results

	ICER vs IPC, AUD per QALY gained	CE QALY simulations vs IPC, %	ICER vs IPC, AUD per VTE avoided	CE VTE simulations vs IPC, %
LMWH	Dominated	1.2	Dominated	0.2
Apixaban	12,656	76.4	3022	46.4
Dabigatran	51,224	55.2	73,824	21.4
Rivaroxaban	55,714	30.8	10,947	10
IPC + LMWH (7 days + 23 days)	Dominated	1.8	Dominated	3.2
IPC + apixaban (7 days + 23 days)	14,000	87.8	4960	36.6

Outcomes and costs of the assessed treatment modalities compared to IPC. ICER vs IPC, AUD per QALY gained: incremental costs to gain one additional QALY; CE QALY simulations vs. IPC: percent of simulations where the comparator was considered more cost-effective than IPC regarding quality of life; ICER vs IPC, AUD per VTE avoided: incremental costs to avoid one additional VTE event; CE VTE simulations vs IPC: percentage of simulations where the comparator was considered more cost-effective than IPC regarding VTE prevention

*AUD* Australian dollars, *CE* cost-effective, *Dominated* more expensive and fewer QALYs accumulated compared to IPC alone, *ICER* incremental cost-effectiveness ratio, *IPC* intermittent pneumatic compression, *LMWH* low-molecular-weight heparin, *QALY* quality-adjusted life year, *VTE* venous thromboembolism

**Fig. 1:**
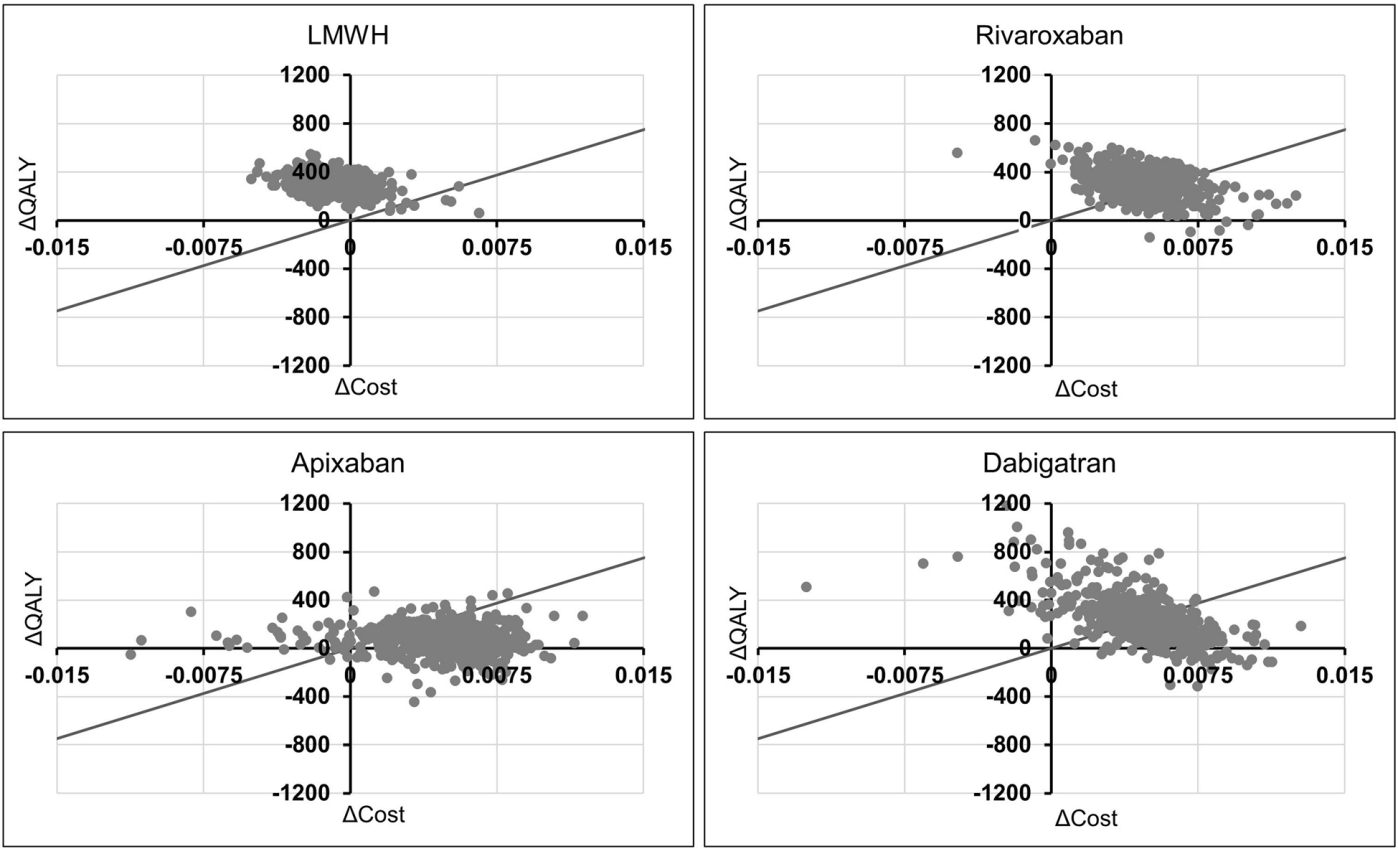
Cost-effectiveness plane for pharmacoprophylaxis versus IPC. Each graph shows the cost-effectiveness plane for one method of VTE prophylaxis when compared to IPC. The change in QALYs (*x*-axis, pharmacoprophylaxis—IPC) is plotted against change in costs (*y*-axis, pharmacoprophylaxis—IPC). For comparative purposes, all graphs have the same axis ranges. Points falling below the diagonal line would be considered cost-effective at a willingness-to-pay (maximum cost that is considered acceptable for payers) threshold of AUD 50,000 per QALY gained

Results were generally similar when the outcome considered was VTE events avoided rather than QALYs gained. One outlier was rivaroxaban, where the ICER was AUD $55,714 per QALY gained and AUD $10,947 per VTE avoided. The difference between the ICERs has demonstrated that although rivaroxaban substantially reduces VTE events, it increases the risk of bleeding which is reflected by the QALY estimate. Sensitivity analyses found that only apixaban and IPC + apixaban were likely to be cost-effective versus IPC alone when considering the cost per QALY gained (Table 2 and Fig. 1). If the focus is on VTE events avoided, then no intervention is expected to be cost-effective versus IPC alone more than 50% of the time.

**Fig. 2:**
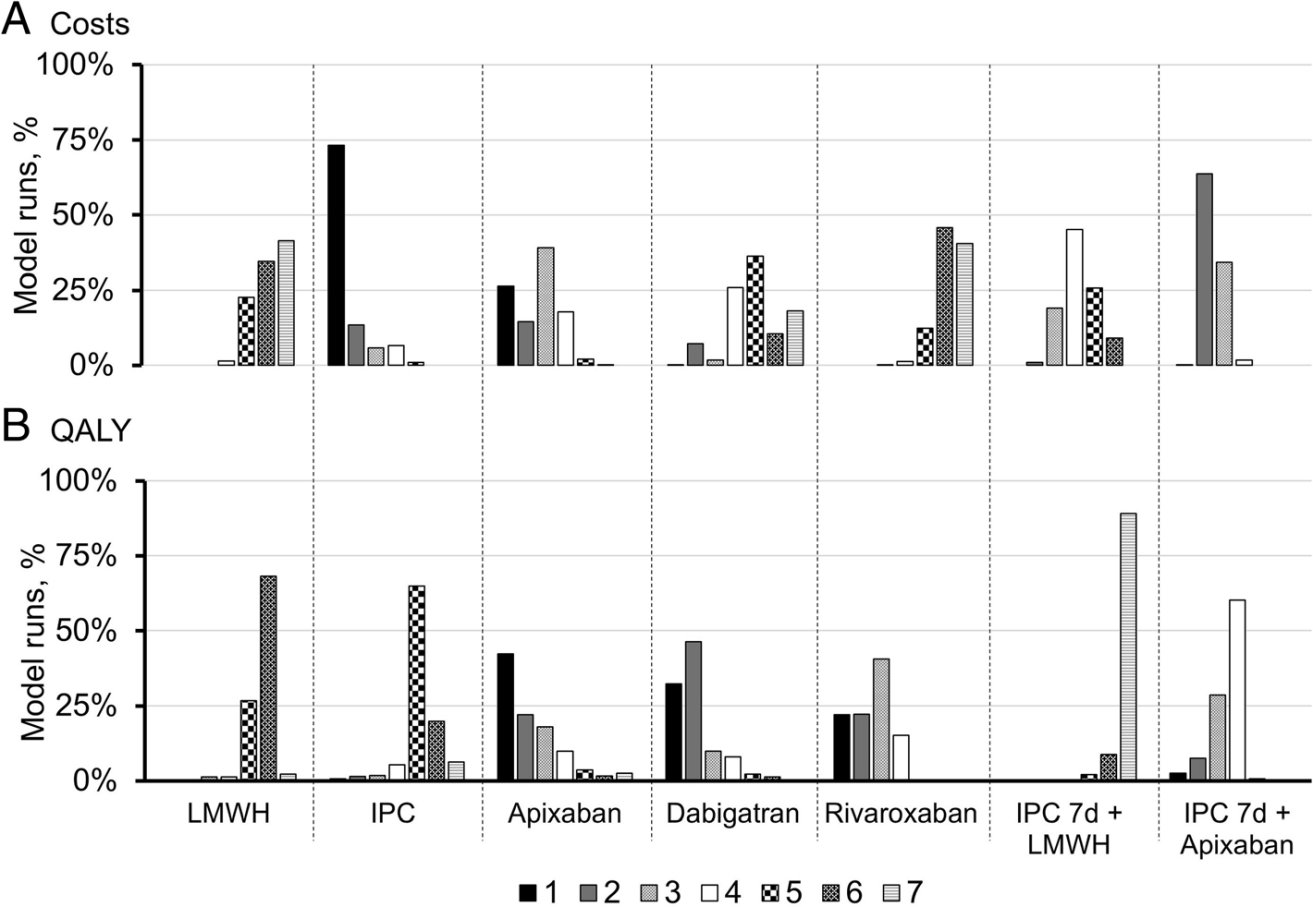
Prophylaxis ranking on costs and QALYs. The ranking distribution per prophylaxis modality is presented for costs (**a**) and QALYs (**b**). The lowest rank (1) is associated with the best outcome: lowest costs or highest QALYs; for rank 7, the outcomes are the worst: highest cost or lowest QALYs

To further investigate the sensitivity of results to changes in input parameters, the outcome for prophylaxis method for each run was ranked from 1 (best) to 7 (worst). The ranking confirmed that in the majority of cases, IPC is expected to have the lowest cost of care (Fig. 2). Apixaban and dabigatran are most likely to result in the best outcomes with respect to QALYs, and they differ in their cost outcomes though with apixaban generally of lower cost and dabigatran generally of higher cost.

**Fig. 3:**
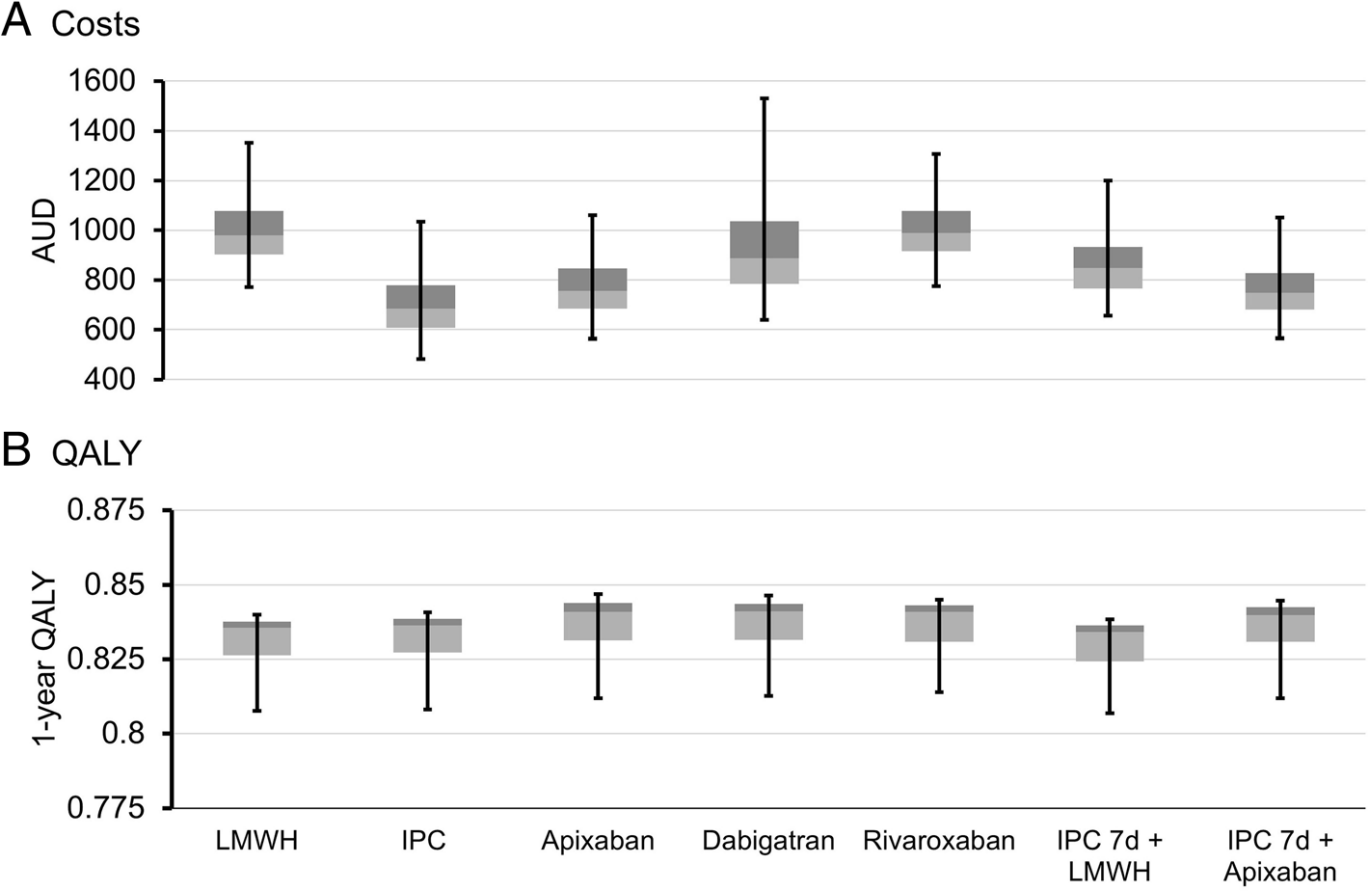
The distribution of costs and QALYs by prophylaxis modality. Boxplots showing the 95% CI as error bars. The light grey box is the 25th to 50th percentile (Q2) and the dark grey the 50th to 75th percentile (Q3). The border between the two is the median value. Results are shown for costs (**a**) and QALYs (**b**)

Most would consider the goal of health care optimization is to provide better therapy at a lower cost. No VTE prophylaxis option at this time can be considered optimal for all patients. The rankings inform on general trends but ignore the magnitude of the difference between outcomes. To assess potential differences in the distributions of costs and QALY attributable to the prophylaxis regimens, the median and interquartile range for each outcome was examined (Fig. 3). While for QALY outcomes, the range was very limited, with the interquartile ranges for all modalities overlapping. The costs had higher fluctuations, with the interquartile range for IPC falling below that of LMWH, dabigatran and rivaroxaban. When assessing the cost drivers, consistently, the bleeding risk and the VTE risk could be identified as main factors for all prophylaxis regimens. As IPC has the lowest risk for minor bleeding and apixaban the lowest risk for VTE, this likely explains the two modalities resulting in the lowest costs of care.

Combination of pharmaco- and mechanical VTE prophylaxis is also in use clinically. After analysis of the multi-national Global Orthopaedic Registry, Warwick et al. reported that 58.1% of THA and TKA patients in the USA used IPC plus LMWH or warfarin [22]. Outside of the USA, this percentage dropped to 11.43% as LWMH alone dominated the prescriptions [22]. The combination of IPC and LMWH has been found through meta-analysis to likely be beneficial, [14, 23], but limited data resulted in wide confidence intervals. Here, we assessed the use of IPC + LMWH and IPC + apixaban. Given that combination data are limited, we considered that IPC will be used in the immediate post-operative period when the risk of bleeding is greatest and then prophylaxis will transition to pharmacological options after day 7. Results indicate that IPC + LMWH is of limited benefit, accumulating fewer QALY_S_ and higher costs compared with most other prophylaxis modalities (Fig. 3). The use of IPC + apixaban, on the other hand, is often one of the lower cost options and has an above average QALY return (Fig. 3). Although unlikely to be cost-effective when measured by the cost per VTE event avoided (Table 2), the combination is the most likely to be cost-effective when considering the cost per QALY gained. This may suggest that it provides a satisfactory balance between preventing early post-operative bleeding and maintaining VTE prophylaxis to 30-days post-operatively.

## Discussion

VTE has been identified as a major problem in Australia, with incidence rates above the average for OECD countries [3]. VTE is particularly common after THA and TKA and is associated with substantial adverse effects on patient outcomes. Anticoagulant VTE prophylaxis is recommended and widely used following THA and TKA, but all anticoagulants have inherent risk of bleeding. It is becoming understood that both from a patient and payer’s perspective, it is pivotal to balance the risk of VTE and bleeding [10, 15]. Until now, discussion and comparison of prophylaxis modalities has focused on clinical efficacy and safety. Healthcare resources are scarce and hence the economics of care—after taking both efficacy and safety simultaneously into account—is of growing importance. This study seeks to examine which of the available VTE prophylactic modalities will be most beneficial and cost-effective to all stakeholders: patients, providers and payers.

In-line with meta-analysis data on safety and efficacy, [24, 25] we found that apixaban showed a better health economic profile than other DOACs. Data for IPC indicates equivalent efficacy and improved safety, [14, 15] which resulted in IPC having the lowest costs of care. Use of IPC may limit patient mobility and reduce their quality of life while it is in use, and this was one factor resulting in IPC, when used for an extended period after surgery, having reduced QALY compared to oral DOACs. The results add to the debate on the most appropriate form of VTE prophylaxis, as it raises the question of how to define cost-effectiveness. Apixaban is likely to be most cost-effective on a cost per QALY basis, but IPC is most cost-effective when considering the cost per VTE event avoided. Furthermore, the minor differences in QALY across all the prophylaxis modalities lead us to question whether a cost minimization approach would be most appropriate as it is likely that neither patients nor providers will consider a QALY difference of 0.004 as substantial or relevant. This difference equates to 1.5 days of perfect quality of life over the year or approximately 2 days of mean quality of life in this patient population. If QALY differences are removed from consideration, IPC, apixaban, or a combination of the two would likely be the most appropriate prophylaxis methods.

There is an ongoing debate about using risk stratification to optimise the allocation of VTE prophylaxis. The aim is to minimise and hopefully eliminate both clinically relevant bleeding and VTE. To this end, patients at high bleeding risk should avoid pharmacoprophylaxis at least in the immediate post-operative period, whereas those at high risk of VTE without a high bleeding risk should receive the most efficacious prophylaxis available. The former patients are often best served to have IPC alone while the latter receive pharmacoprophylaxis in conjunction with mechanical prophylaxis. Those at high risk of both bleeding and VTE are problematic from this perspective, but a combination of IPC + apixaban where patients initiate on IPC alone and transition to apixaban once their bleeding risk has subsided may provide the best compromise in this population. Well-designed clinical studies in this setting would help inform the hypothesis developed from our analysis. The three options we identify as cost-effective can thus meet the needs of a risk-stratified, individualised approach to VTE prophylaxis.

Given reports of high VTE rates in Australia [3], VTE pharmacoprophylaxis has remained as the main focus of clinical interest. Recent studies have indicated VTE rates in Australia range from 0.7% (in-hospital) to 4.7%, [26, 27] almost comparable to the rates of bleeding (1.5–6.7%) [27, 28]. Although the generalisability of these figures remains uncertain, it does speak to the issue raised by Campbell et al. that bleeding and adverse events were seemingly under-emphasised in pharmacoprophylaxis studies [29]. This issue was revisited by Miller et al., in 2016, who found that although pharmacoprophylaxis prevented VTE deaths, there was a net increase in deaths due to bleeding [30]. When considering post-THA and TKA care, finding the optimal balance between VTE prevention and risk of bleeding for each individual patient is a challenging but necessary task for the treating physician. Our results suggest that individualised approach to VTE prophylaxis in the immediate post-surgical period—using IPC, apixaban or a combination of both—is most cost-effective in reducing VTE for most patients after THA or TKA without compromising their quality of life compared to alternative strategies.

This economic analysis has several limitations. First, we have not considered the use of aspirin alone compared to LMWH, IPC, DOACs or a combination of these strategies. Second, our analysis was based on average benefit and safety data and may not be applicable to patients with significant comorbidities which may increase their VTE and bleeding risks. Finally, the costs incurred by DOACs are likely to reduce with time when their patent periods have expired which would have significant effect on their future cost-effectiveness.

## Conclusion

It is likely that the most cost-effective method of VTE prophylaxis is apixaban or IPC, or a combination of the two—either simultaneously or sequentially. The choice of VTE prophylaxis should be informed by the relative risk of VTE and bleeding in relation to surgery. IPC would be of most benefit immediately after surgery when patients are most at risk of bleeding. Apixaban plays an important role when the bleeding risk has subsided and patients are mobile. For patients at high risk of VTE or with an extended hospital stay, a combination of IPC and apixaban during the entire hospital stay should be seriously considered.

## Abbreviations

CI: Confidence interval
CrI: Credible interval
DOACs: Direct oral anticoagulants
DVT: Deep vein thrombosis
EQ-5D: EuroQoL 5-dimensions
HIT: Heparin-induced thrombocytopenia
ICH: Intracranial haemorrhage
IPC: Intermittent pneumatic compression
LMWH: Low-molecular-weight heparin
OECD: Organisation for Economic Co-operation and Development
PE: Pulmonary embolism
PTS: Post-thrombotic syndrome
QALYs: Quality-adjusted life years
THA: Total hip arthroplasty
TKA: Total knee arthroplasty
VTE: Venous thromboembolism
WHO: World Health Organization

## References

[1] National Health and Medical Research Council. Preventing venous thromboembolism in hospitalised patients: Summary of NHMRC activity 2003–2010. Melbourne: National Health and Medical Research Council; 2011.

[2] Australian Institute of Health and Welfare (2009). Towards national indicators of safety and quality in health care.

[3] Australian Institute of Health and Welfare (2016). OECD health-care quality indicators for Australia 2015.

[4] Jha AK, Larizgoitia I, Audera-Lopez C, Prasopa-Plaizier N, Waters H, Bates DW (2013). The global burden of unsafe medical care: analytic modelling of observational studies. BMJ Qual Saf.

[5] Australian Commission on Safety and Quality in Health Care. Venous Thromboembolism Prevention Clinical Care Standard. Sydney: ACSQHC; 2018.https://www.safetyandquality.gov.au/our-work/clinical-care-standards/venous-thromboembolism-prevention-clinical-care-standard/. Accessed 20 Mar 2019.

[6] Peel TN, Cheng AC, Liew D, Buising KL, Lisik J, Carroll KA (2015). Direct hospital cost determinants following hip and knee arthroplasty. Arthritis Care Res (Hoboken).

[7] Duff J, Walker K, Omari A, Stratton C (2013). Prevention of venous thromboembolism in hospitalized patients: analysis of reduced cost and improved clinical outcomes. J Vasc Nurs.

[8] Australian Orthopaedic Association National Joint Replacement Registry. Reported hip procedures. https://aoanjrr.sahmri.com/hips. Accessed 22 June 2018.

[9] Australian Orthopaedic Association National Joint Replacement Registry. Reported knee procedures. https://aoanjrr.sahmri.com/knees. Accessed 22 June 2018.

[10] Noel SE, Alasdair Millar J (2014). Current state of medical thromboprophylaxis in Australia. Australas Med J.

[11] National Health and Medical Research Council. Clinical practice guideline for the prevention of venous thromboembolism (deep vein thrombosis and pulmonary embolism) in patients admitted to Australian hospitals. Melbourne: National Health and Medical Research Council; 2009. 10.1111/j.1445-5994.2012.02808.x 22697152

[12] ANZ Working Party on the Management and Prevention of Venous Thromboembolism. Prevention of venous thromboembolism: best practice guidelines for Australia & New Zealand, 4th edn. 2007.

[13] Gallagher M, Oliver K, Hurwitz M (2009). Improving the use of venous thromboembolism prophylaxis in an Australian teaching hospital. Qual Saf Heal Care.

[14] Ho KM, Tan JA (2013). Stratified meta-analysis of intermittent pneumatic compression of the lower limbs to prevent venous thromboembolism in hospitalized patients. Circulation..

[15] Sharfman ZT, Campbell JC, Mirocha JM, Spitzer AI (2016). Balancing thromboprophylaxis and bleeding in total joint arthroplasty: impact of eliminating enoxaparin and predonation and implementing pneumatic compression and tranexamic acid. J Arthroplast.

[16] Riva N, Bellesini M, Di Minno MND, Mumoli N, Pomero F, Franchini M (2014). Poor predictive value of contemporary bleeding risk scores during long-term treatment of venous thromboembolism. Thromb Haemost.

[17] Saunders R, Comerota AJ, Ozols A, Torrejon Torres R, Ho KM (2018). Intermittent pneumatic compression is a cost-effective method of orthopedic postsurgical venous thromboembolism prophylaxis. Clinicoecon Outcomes Res.

[18] O’Connell S, Bashar K, Broderick BJ, Sheehan J, Quondamatteo F, Walsh SR (2016). The use of intermittent pneumatic compression in orthopedic and neurosurgical postoperative patients. Ann Surg.

[19] Gómez-Outes A, Terleira-Fernández AI, Suárez-Gea ML, Vargas-Castrillón E (2012). Dabigatran, rivaroxaban, or apixaban versus enoxaparin for thromboprophylaxis after total hip or knee replacement: systematic review, meta-analysis, and indirect treatment comparisons. BMJ..

[20] Cohen AT, Hamilton M, Mitchell SA, Phatak H, Liu X, Bird A (2015). Comparison of the novel oral anticoagulants apixaban, dabigatran, edoxaban, and rivaroxaban in the initial and long-term treatment and prevention of venous thromboembolism: systematic review and network meta-analysis. PLoS One.

[21] Haac BE, O’Hara NN, Mullins CD, Stein DM, Manson TT, Johal H (2017). Patient preferences for venous thromboembolism prophylaxis after injury: a discrete choice experiment. BMJ Open.

[22] Warwick D, Friedman RJ, Agnelli G, Gil-Garay E, Johnson K, FitzGerald G (2007). Insufficient duration of venous thromboembolism prophylaxis after total hip or knee replacement when compared with the time course of thromboembolic events: findings from the Global Orthopaedic Registry. J Bone Jt Surg - Br.

[23] Pavon JM, Adam SS, Razouki ZA, McDuffie JR, Lachiewicz PF, Kosinski AS (2016). Effectiveness of intermittent pneumatic compression devices for venous thromboembolism prophylaxis in high-risk surgical patients: a systematic review. J Arthroplast.

[24] Cohen A, Drost P, Marchant N, Mitchell S, Orme M, Rublee D (2012). The efficacy and safety of pharmacological prophylaxis of venous thromboembolism following elective knee or hip replacement: systematic review and network meta-analysis. Clin Appl Thromb.

[25] Dequen P, Sutton AJ, Scott DA, Abrams KR. Searching for Indirect Evidence and Extending the Network of Studies for Network Meta-Analysis: Case Study in Venous Thromboembolic Events Prevention Following Elective Total Knee Replacement Surgery. Value Heal. 2014;17(4):416–23. doi:10.1016/j.jval.2014.02.013. 10.1016/j.jval.2014.02.013 24969002

[26] Mirkazemi C, Bereznicki LR, Peterson GM (2013). Thromboprophylaxis following hip and knee arthroplasty. Intern Med J.

[27] Pow RE, Vale PR (2015). Thromboprophylaxis in patients undergoing total hip and knee arthroplasty: a review of current practices in an Australian teaching hospital. Intern Med J.

[28] King DAL, Pow RE, Dickison DM, Vale PR (2016). Apixaban versus enoxaparin in the prevention of venous thromboembolism following total knee arthroplasty: a single-centre, single-surgeon, retrospective analysis. Intern Med J.

[29] Campbell D, Smith P, Lewis P, Bruce W (2010). Controversies of thrombophylaxis following knee arthroplasty surgery. ANZ J Surg.

[30] Millar JA, Gee ALK (2016). Estimation of clinical and economic effects of prophylaxis against venous thromboembolism in medical patients, including the effect of targeting patients at high-risk. Intern Med J.

